# Insights into the Metabolism, Disposition, and Quantitative Profile of mGlu5 NAM AE90015 with Metabolite Identification and a Novel Integration Method

**DOI:** 10.3390/molecules29235724

**Published:** 2024-12-04

**Authors:** Zhiyang Zack Zou, Ming-Jie Han, Yu Chang, Guiying Li

**Affiliations:** 1 Department of DMPK & TOX, Global Health Drug Discovery Institute, Zhongguancun Dongsheng International Science Park, Beijing 100192, China; hanmingjie_2024@outlook.com (M.-J.H.); yu.chang@ghddi.org (Y.C.); 2 TB Alliance, 80 Pine St. 20th Floor, New York, NY 10005, USA; guiying.li@tbaliance.org

**Keywords:** metabolism, disposition, quantification, excretion, glucuronidation

## Abstract

AE90015 is a highly specific and effective negative allosteric modulator (NAM) for the human mGlu5 receptor, showing significant promise for treating Parkinson’s disease. An in vivo rat oral dose study was conducted on AE90015, which involved the collection of urine and bile samples over a 24 h period. At the study’s endpoint, plasma, liver, brain, and renal tissues were also collected. A total of 30 metabolites of AE90015 were identified and structurally characterized or detected using high-resolution LC-MS/MSn. These metabolites fall into four categories: mono-hydroxyl, di-hydroxyl, mono-hydroxyl glucuronide, and di-hydroxyl glucuronide. This study provided a comprehensive overview of the metabolism, excretion, and disposition of AE90015, a promising NAM. The primary clearance pathway for AE90015 is mono-oxidation, accounting for 96% of the total, while direct excretion via renal and bile routes accounted for only 0.5%. Bile emerged as the predominant excretion route, at 65%, for metabolites and a minor amount of parent compound, which contrasts with the common assumption that urine would be the primary excretion pathway, which accounted for 26%. Each adamantyl and pyrazine moiety of AE90015 undergoes a one-time oxidation, while the pyridyl portion remains unmetabolized. Secondary metabolites, such as di-hydroxylated forms and glucuronide conjugates, do not contribute to clearance. In this work, a new quantification method combining UV and mass spectra integration was developed, allowing for the quantification of overlapping metabolite peaks. This novel approach proved to be highly effective for metabolite identification in early preclinical studies.

## 1. Introduction

Metabotropic glutamate receptor 5 (mGlu5) is predominantly expressed by post-synaptic excitatory terminals and astrocytes within the central nervous system (CNS) [1,2]. Its structural composition includes seven transmembrane-spanning domains and an extracellular N-terminal domain. The transmembrane region harbors an allosteric binding site, while the extracellular domain hosts an orthosteric binding site. Through its coupling with Gq proteins, mGlu5 can activate signaling pathways and is notably associated with conditions such as anxiety, mood disorders, and pathological pain states. mGlu5 can activate signaling pathways and is notably associated with conditions such as anxiety, mood disorders, and pathological pain states [3,4,5]. The anatomical distribution of mGLu5 is notably similar in rodents, non-human primates, and humans. The negative allosteric modulation (NAM) mechanism of mGlu5 has been subject to extensive investigation in the preclinical scientific literature and has demonstrated significant therapeutic potential in proof-of-concept preclinical studies [6,7,8]. Notably, mGlu5 NAM is considered to be a promising avenue for therapeutic intervention in conditions like Parkinson’s disease [9,10,11], Alzheimer’s disease [12,13,14], neuropathic inflammatory [15,16], and migraine pain [17], as well as Fragile X syndrome [18,19].

AE90015 is a highly specific negative allosteric modulator (NAM) for the human mGlu5 receptor, exhibiting an impressive affinity of 18.5 nM [6]. It has been demonstrated that AE90015 has a decent selectivity for and binds to mGlu5, showing no significant efficacy or affinity for other mGlu receptors, including mGlu1, mGlu2, mGlu3, mGlu4, and mGlu7. Due to its valuable biological properties, AE90015 has undergone a series of ADME studies. This research aims to address two key questions: firstly, whether the primary clearance pathway of AE90015 in rats is through metabolism or direct excretion, and secondly, if metabolism is the major route, what metabolites are produced, and where are they distributed or excreted; this is also related to toxicity and efficacy. We conducted both in vitro and in vivo experiments in rats, including bile and urine collection. A sophisticated metabolite identification was carried out using high-resolution LC-MS/MSn and the total AE90015 and its metabolites were quantified using a novel UV and mass spectrum integration. Though AE90015 did not eventually become a preclinical candidate, this study offers an interesting sketch of the metabolism, excretion, and disposition of AE90015 as a typical Class II compound.

## 2. Results and Discussion

### 2.1. Initial Metabolite Identification and Structure Elucidation of AE90015 and Metabolites in Rat Liver Microsome

Metabolite identification and quantification were carried out using high-resolution LC-MS/MSn and UV measurements. The incubation of AE90015 from a rat liver microsome used the following conditions: 25 µM AE90015 in 0.5 mg/mL rat liver microsome incubated at 37 °C for 0 and 60 min. During the microsome incubation, only hydroxyl and di-hydroxyl metabolites were observed or produced. The di-hydroxyl metabolites originated from the mono-hydroxyl metabolites; they were secondary metabolites. AE90015 is cleared whenever mono-hydroxyl metabolites are produced; however, not all mono-hydroxyl metabolites could be detected in this study. This is because some mono-hydroxyl metabolites were further oxidized to di-hydroxyl metabolites. To accurately account for the metabolic clearance of AE90015, we must consider both the mono-hydroxyl metabolites and all of the secondary metabolites.

**Figure 1:**
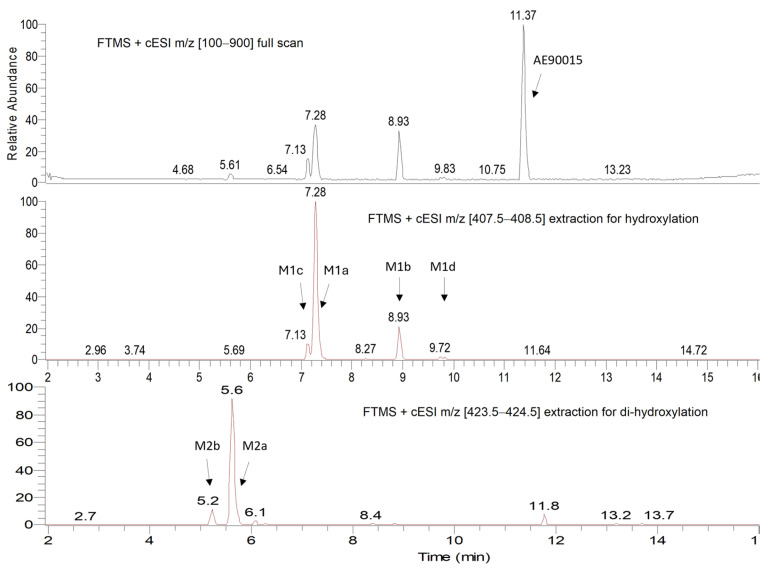
The AE90015 and metabolites in an LC-MS/MSn chromatograph for the human and rat liver microsome incubations. Upper: full mass scan; middle: mass extraction of mono-hydroxy metabolites; lower: mass extraction of di-hydroxy metabolites. M1a, M1b, M1c, and M1d are mono-hydroxy metabolites of AE90015. M1a and M2b are di-hydroxyl metabolites of AE90015.

**Figure 2:**
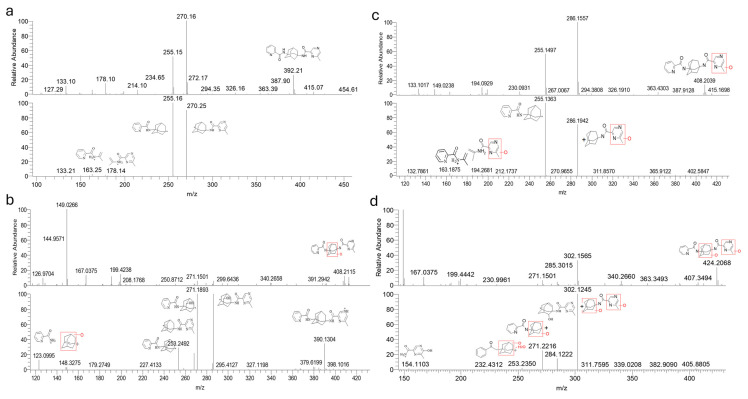
Elucidation of the structures of AE90015 and its metabolites using a high-resolution, accurate LC-MS/MSn-data-dependent scan of MS, MS2, and MS3. Accurate drug fragmentation mass assignments based on the rules derived from organic chemistry and mass collision of organic compounds were achieved for (**a**) AE90015; (**b**) adamantane hydroxylation metabolites, such as M1e; (**c**) methyl-pyrazine hydroxylation metabolites such as M1b; and (**d**) methyl-pyrazine and adamantane di-hydroxylation metabolites, such as M2b. With powerful high-resolution and accurate mass spectrometry and the ion trap MS scan and data-dependent scans of MS2 and MS3, the elucidation of the structure was made relatively easy and more accurate. Figure 2 exemplifies the structures elucidated in this research.

Figure 1 and Figure 2 illustrate the retention time of each metabolite in the UPLC profile after incubation. A total of eight different metabolites were identified in the LC-MS/MSn spectrum. Two of these metabolites, M1d and M1b, exhibited retention times of 8.9 min and 9.8 min, respectively, representing the mono-oxidation of the pyrazine portion, as the elucidations of the molecular weights and structures of their fragmentations in the mass spectra proved. Furthermore, four mono-oxidation metabolites were identified on the adamantyl portion, M1a, M1c, M1f, and M1e, with retention times at 7.3 min, 7.1 min, 8.3 min, and 8.3 min, respectively. Lastly, two metabolites, M2a and M2b, experienced di-hydroxilations on both adamantyl and pyrazine residues, with retention times of 5.2 min and 5.6 min. The primary biotransformation of AE90015 during microsomal incubation was notably mono-oxidation on the adamantyl portion. Additionally, the levels of all metabolites increased with prolonged incubation, highlighting the dynamic nature of AE90015′s metabolic biotransformations.

1. Metabolite identification, structure elucidation, and quantification of metabolites in plasma, urine, and bile from rats in an in vivo study.

### 2.2. In Vivo Urine, Bile, Plasma, and Tissue Sample Collection from Rats

The in vivo rat studies were performed following the administration of a 30 mg/kg dose of AE90015. Plasma, urine, and tissue samples were collected from two rats in a metabolic cage, and bile samples were collected from two bile duct-cannulated rats. The urine and bile samples were collected from 0 to 24 h post-administration. The tissues and plasma samples were collected at 24 h post-administration. This study was carried out to explore all metabolites, particularly the phase II metabolites, and to identify the metabolites being excreted and understand their excretion pathways. Additionally, determining whether AE90015 itself is directly cleared through urine and bile is crucial for explaining its pharmacokinetic clearance value in rats.

### 2.3. UPLC-MS/MSn Analysis

To identify the metabolites, we employed UPLC-MS/MSn with an LTQ-Orbitrap high-resolution mass spectrometer (Thermo Fisher Scientific, Waltham, MA, USA), which facilitated reliable structure elucidation. Metabolite identification and structural elucidation were achieved through a combination of fragmentation and accurate mass data analysis, providing a detailed representation of the metabolites’ chemical structures.

### 2.4. UPLC-UV Analysis and UV to Mass Response Ratio for Quantification

For quantification, a photodiode array detector connected to UPLC in parallel to the LTQ-Obitrap mass spectrometer, detecting at 260 nm absorption, and combined with mass response was used to quantitatively measure, sometimes with compromised accuracy, the metabolites and AE90015 in all of the samples. For well-separated metabolites in the liquid chromatogram, their UV 260 nm absorption peak integration represented their molar response. When the metabolites’ UV peaks overlap with each other, UV integration is not feasible, but the integration of extracted metabolite mass is much easier. However, the mass response of each metabolite does not accurately represent its molar response while UV absorption does. It was assumed that each metabolite’s UV integration-to-mass integration ratio is equal, allowing for a good approximation within the same category of metabolites. This assumption was validated by many of the metabolites measured in this study and previous studies. When no UV integration is available, multiplying the mass integration by the ratio of the mass to the UV response yielded in the UV integration.

This comprehensive approach enabled a quantitative assessment of all metabolites and AE90015 concentrations in the plasma, urine, bile, and tissue samples.

### 2.5. Structure Elucidation of AE90015 Phase I and Phase II Metabolites

**Figure 3:**
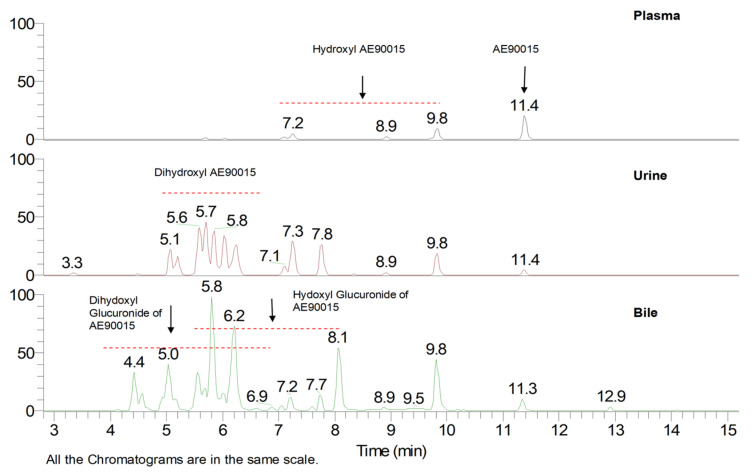
LC-MS chromatograms of AE90015 and its metabolites in pooled plasma, urine, and bile from bile duct-cannulated rats 24 h following the administration of an oral dose of 30 mg/kg of AE90015 to the rats. The red dotted line indicates the retention time range of those metabolites.

**Figure 4:**
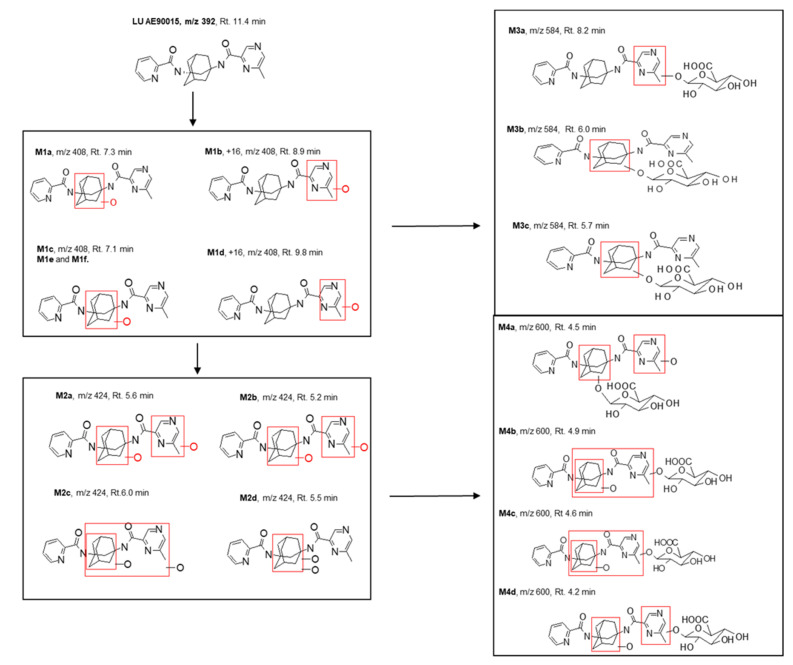
The elucidation of the structures of the glucuronide metabolites of AE90015 based on fragmentations of the mass spectrometry analyses of the plasma, urine, and bile samples. Red rectangles indicates the hydroxylation positions.

We conducted experiments with bile duct-cannulated rats and a metabolic cage to assess the roles of biliary and renal excretion. The profiling of the metabolites in plasma, urine, and bile samples was performed using UPLC-MS, as illustrated in Figure 3, and these metabolites were subsequently identified through an MS/MSn analysis. AE90015 experienced mono-hydroxylation and subsequently di-hydroxylation during phase I metabolism, and then conjugated with the hydroxyl to generate the hydroxyl-glucuronide and di-hydroxyl-to-di-hydroxyl glucuronide metabolites during phase II metabolism. The mono-hydroxylation happened in two distinct phases: pyrazine oxidation, giving rise to M1b (8.9 min) and M1d (9.8 min), and adamant oxidation, leading to M1a (7.3 min), M1c (7.1 min), M1f (8.5 min), and M1e (8.3 min), which are distinguished at different oxidation sites during pyrazine or adamant oxidation, but not on pyridyl sites. The di-hydroxylated metabolites of AE90015 were structurally elucidated through one pyrazine oxidation and one adamant oxidation, yielding M2a (5.6 min) and M2b (5.2 min). Other minor metabolites were identified by their categories but their exact structures were elucidated due to the presence of weak mass signals, which include mono-hydroxylation metabolites M1g (9.9 min), di-hydroxylation with M2c (6.0 min), M2d (5.5 min), M2e (5.0 min), and M2f (6.7 min). Notably, all of the mono-hydroxylated AE90015s were eluted within a retention time from 7.2 min to 9.9 min. Meanwhile, the di-hydroxylated AE90015 exhibited increased polarity and were eluted between 5 min and 6 min. Furthermore, the phase II metabolites included mono-glucuronides with elution times ranging from 4 min to 7 min, and di-hydroxyl glucuronides of AE90015 eluted at retention times in the range of 5.5 min to 8.1 min. The metabolites and parent compound AE90015 can be ranked according to their retention times in the reverse-phase chromatography as follows: parent drug AE90015, mono-hydroxyl metabolites, mono-hydroxyl glucuronides, di-hydroxyl metabolites, and di-hydroxyl glucuronides. Although the mono-hydroxyl glucuronide metabolites contain three hydroxyl groups and one carbonyl group more than the di-hydroxyl metabolites, making them more polar than the di-hydroxyl metabolites, the retention time of the di-hydroxyl metabolites is shorter (Figure 4). This difference is because retention time is not only influenced by lipophilicity but also by molecular weight, with glucuronides having a higher molecular weight than their di-oxidation counterparts.

**Figure 5:**
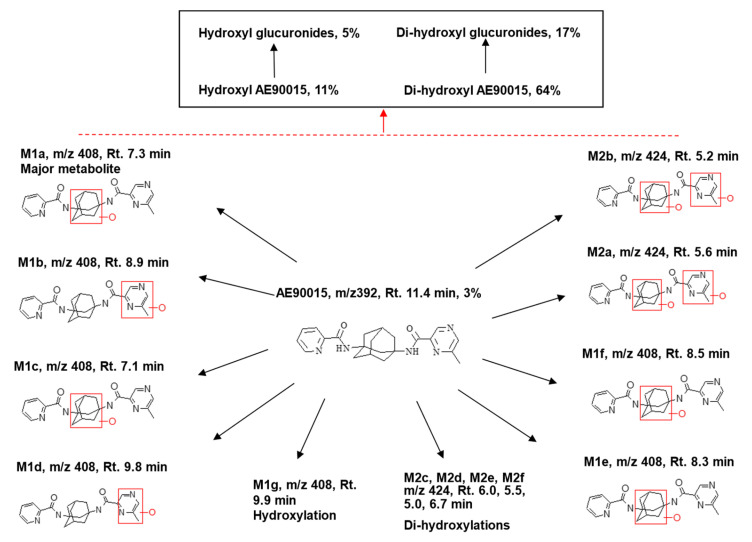
Summary of all metabolites identified in the rat in vitro liver microsomes and in vivo samples, including plasma, urine, bile, and tissues. The blank and red arrows indicate the biotransformation directions. The red dashed line includes all of the lower part metabolites.

The careful mass spectrometry elucidation of the structures confirmed that no glucuronide directly conjugated to AE90015 was found. The glucuronidation happened only on hydroxyl groups (Figure 5).

Overall, the parent drug AE90015 and four other categories of metabolites were identified: mono-hydroxyl metabolites, di-hydroxyl metabolites, mono-hydroxyl glucuronides, and di-hydroxyl glucuronides. The total number of metabolites detected was as high as 30, which was related to a moderately high AE90015 clearance of 38 mL/min/kg in rat PK during an in-house experiment. Our structural elucidation confirmed that the phase II metabolites are secondary metabolites. All the di-hydroxy metabolites and glucuronide metabolites are secondary metabolites that do not contribute to the clearance of the parent drug. Once an AE90015 molecule is converted into a mono-hydroxy metabolite, it is cleared. This is why, in vitro–in vivo extrapolation (IVIVE) using microsomal clearance (phase I metabolism) often correlates well with vivo metabolic clearance [20].

### 2.6. Insight into AE90015 and All Its Metabolite Quantifications

The distribution of normalized integration values for the parent drug AE90015 and its metabolites in plasma, urine, bile, and tissues, including liver, brain, and kidney, is summarized in Table 1. Notably, the metabolite levels in circulating blood were remarkably low. In this experiment, all amounts of AE90015 and its metabolites in the plasma, urine, and bile samples were obtained from UV responses, which were adjusted to their total volume in the rat. For the liver, brain, and kidney tissues, these amounts were normalized to tissue weight. The sample dilution factors used in the analysis were also converted back.

The values in Table 1 represent the relative molar amounts of each metabolite of AE90015 remaining in the plasma and tissues or excreted in urine or bile 24 h post-dose. These values were derived either directly from UV 260 nm integration or from mass response integration, multiplied by the UV/mass ratio for each metabolite category, and further adjusted to plasma, urine volume, or tissue weight. The details of the calculation methods are provided in Section 3.

AE90015 and its phase I metabolites were detected in all analyzed tissues. However, glucuronide metabolites were not found in plasma, liver, kidney, or brain, indicating that hydroxyl glucuronides and di-hydroxyl glucuronides are true excretion metabolites, with the majority being eliminated through bile. As these glucuronides are absent in the body, they are unlikely to demonstrate any biological activity.

Bile and urine analyses showed that only small amounts of AE90015 were excreted in urine and bile. This suggests that bile and urine excretion contribute minimally to the clearance of the parent drug, in line with the established fact that drug metabolism is the primary pathway for the clearance of most drugs [21,22]. The largest quantities of metabolites were excreted in bile, particularly glucuronide metabolites, including mono-hydroxy and di-hydroxy glucuronides. This indicates that bile is the main route for glucuronide elimination, while urine serves as an important pathway for excreting hydroxyl and di-hydroxyl metabolites, but not glucuronides. The larger molecular weight of glucuronide metabolites may reduce their filtration through the kidneys.

**Figure 6:**
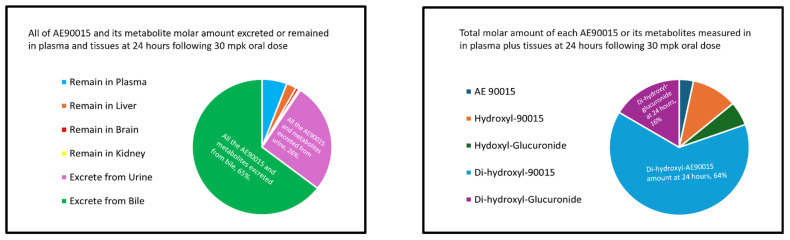
Total molar amounts of AE90015 and its metabolites at various time points in the 24 h following the administration of 30 mg/kg to 2 rats.

The chart on the left side of Figure 6 clearly demonstrates that most AE90015-related species are excreted in bile, including glucuronide metabolites and a higher quantity of mono-hydroxy and di-hydroxy metabolites compared to urine. The chart on the right side of Figure 6 reveals that di-hydroxy metabolites were the most abundant metabolites of AE90015, followed by di-hydroxy glucuronide metabolites. To accurately account for all the AE90015 cleared, we must account for all of the metabolites, including mono-hydroxyl, as well as di-hydroxyl and mono-hydroxyl glucuronide and di-hydroxyl glucuronide metabolites, because all of them represent a mono-hydroxylation.

## 3. Materials and Methods

The compound AE90015, molecular weight 391.2, was synthesized at Lundbeck Research (Deerfield, IL, USA). All reagents, including specific cytochrome P450 (P450) inhibitors, were procured from Sigma-Aldrich (St. Louis, MO, USA). Solvents employed for LC-MS/MS met analytical-grade standards. Hepatocytes were obtained from CellzDirect (Austin, TX, USA), while liver microsomes were sourced from XenoTech, LLC (Kansas City, KS, USA).

### 3.1. Incubation of AE90015 with Rat and Human Liver Microsomes Involved the Following Procedures

Liver microsomes from male rats and humans, each at a concentration of 0.5 mg/mL, were incubated with AE90015 at 25 μM. This incubation took place at 37 °C in a potassium phosphate buffer (100 mM, pH = 7.4) containing 3 mM MgCl_2_. The incubations were carried out in the presence of an NADPH regeneration system, which consisted of D-glucose-6-phosphate at 5 mM, β-Nicotinamide adenine dinucleotide phosphate sodium salt hydrate (NADP) at 1 mM, and Glucose-6-phosphate Dehydrogenase from baker’s yeast (G6PDH) at a concentration of 1 U/mL. The incubation times were set to 30 min and 60 min. The reactions were initiated by the addition of NADP and were subsequently terminated by adding a 2-fold-higher amount of chilled acetonitrile. As for the controls, reaction mixtures without microsomes and without NADPH were incubated for 0, 30, and 60 min. After using the acetonitrile for protein precipitation and centrifugation, the generated samples were subjected to analysis and metabolite identification using LC-MS/MSn/DAD after centrifugation.

### 3.2. Bile Duct-Cannulated Surgery and Bile Collections

Two rats weighing 0.266 and 0.252 kg received 30 mg/kg oral doses of AE90015, formulated with hydroxypropyl-beta-cyclodextrin, for the bile collection study, which was carried out for 0–24 h, and a total of 33.6 mL of bile was pooled together from the 2 rats. The two rats used for the bile collection study underwent bile duct cannulation surgery [2,3] 2 days before the AE90015 dosing and bile collection. Before the surgery, the rats were anesthetized with ketamine (43 mg/kg body weight) and xylazine (8.7 mg/kg body weight). After surgical skin preparation, two catheters (intestinal and biliary) were passed subcutaneously from the dorsal scapular region into the abdomen. Through ventral midline abdominal incision, the bile duct was isolated and ligated, and the biliary catheter was inserted into the bile duct and secured. The intestinal catheter tip was placed into the duodenum just distal to the entry point of the bile duct and secured by the placement of a purse string suture. The two catheters were exteriorized at the scapular incision and joined via the use of a rigid cannula to create an uninterrupted loop, allowing the bile to flow from the liver to the intestinal lumen (Absorption Systems, Philadelphia, PA, USA). After dosing by temporarily disconnecting the external loop and collecting free-flowing bile, the volume was determined. After each collection session, the bile duct end and the duodenal end of the catheter were reconnected with a metal connector so that the bile enterohepatic circulation was reestablished.

### 3.3. Rat Urine, Plasma and Tissue Collections Collection

Two male rats, weighing 0.249 and 0.250 kg, were raised in one metabolic cage. Their urine was collected from 0 to 24 h following a 30 mg/kg oral dose of AE90015, formulated with hydroxypropyl-beta-cyclodextrin. The urine from the 2 rats was pooled together for volume measurement and sample analysis. The urine was filtrated for direct injection into a mass spectrometer.

After a 24 h interval following the administration of AE90015, plasma samples and tissues that included intact brains, livers, and kidneys, were harvested. The plasma and the tissues were pooled together. All the intact organs collected were weighed and used to calculate the normalized AE90015 and the molar amounts of its metabolites in the organs. To eliminate any superficial blood contaminants, all the tissue samples were rinsed with ice-cold 0.9% NaCl. Following the rinsing procedure, the tissue samples were weighed and subsequently homogenized using a tissue homogenizer in a physiological saline solution at a ratio of 1:4 (*w/v*). After homogenization, the samples were subjected to centrifugation at 3000× *g* for 10 min, and the resulting supernatant was added to 3-fold of acetonitrile and the mixture was then centrifuged again at 3000× *g* for 10 min; the supernatant was analyzed using an LC-MS/MS/DAD system. Metabolite identification and quantification was conducted as well as brain-to-plasma ratio (B/P ratio) estimation.

### 3.4. LC-MS/MSn/UV Analysis

The equipment used included an Ultra-Performance Liquid Chromatography (UPLC) system (Waters, Milford, MA, USA) coupled with a LTQ Orbitrap mass spectrometer (Thermo Fisher Scientific, Waltham, MA, USA), capable of high-resolution mass scans (FTMSs). Additionally, a Photodiode Array (PDA) detector (scan range 254–319 nm) (Waters, Milford, MA, USA) was connected in parallel to the mass spectrometer.

Chromatographic separation was performed on a Waters BEH C18 column (1.7 µm, 2.1 × 100 mm) at 40 °C with a flow rate of 0.4 mL/min over a 20 min gradient and UPLC time. The mobile phase consisted of the following phases. Mobile phase A used water with 10 mM of NH4OAc and 0.1% formic acid. Mobile phase B used acetonitrile with 0.1% of formic acid. The elution gradient began with 5% mobile phase A and linearly increased to include 95% mobile phase B over 18 min. The LTQ Orbitrap mass spectrometer from Thermo Fisher Scientific was employed for detection, utilizing high-resolution FTMSs for accurate mass measurements. A positive 4.7 kilovolt MS1 scan and data-dependent ion trap MS2 and MS3 scans with an 18–28 eV collision energy were conducted to elucidate the drug fragmentation structures. To detect glucuronides, sulfates, and glutathione conjugates, a data-dependent neutral loss strategy was used to specifically monitor for neutral losses of 176, 194, 129, 80, and 75.

By using the Waters Photodiode Array (PDA) detector (Waters, Milford, MA, USA) scanning within the range of 254–319 nm, the 260 nm UV light was found to be suitable for detecting and running an integration for the quantification of the metabolites and AE90015 since this wavelength has a high compound response and low rat sample biomatrix interference. In the actual integration of each metabolite and AE90015 to detect a molar response, the first step is to find the well-separated UV peaks for each category of metabolite and AE90015 and carry out UV integration. The second step is to integrate the extracted mass peaks corresponding to those UV peaks. Then, the UV integration of each peak can be divided by its extracted mass peaks to obtain the ratio (R) of each category. We used 4 categories of metabolites which allowed us to find the ratios for hydroxyl AE90015, di-hydroxyl AE90015, hydroxyl glucuronide AE90015, and di-hydroxyl glucuronide AE90015. The final quantification was performed in this way: All the peaks were running integrations of extracted mass peaks. Each integration was multiplied by the UV-to-mass ratio (R) of that category of metabolite. The integration of AE90015 is the same. This procedure allows for a reliable assessment of metabolite levels required for a molar response.

## 4. Conclusions

mGlu5 NAM AE90015, a typical class II compound, underwent a detailed in vitro and in vivo metabolite identification and quantification study in rats. The mono-hydroxylation of AE90015 was found to be the primary metabolic clearance pathway, accounting for nearly the entire clearance of the compound. AE90015 was directly excreted in urine and bile in negligible amounts. This also explains the rationale for using liver microsomal clearance (no phase II metabolism) to calculate IVIVE. Di-hydroxylation, hydroxy glucuronidation, and di-hydroxy glucuronidation did not contribute to its clearance. The true excretory metabolites were the mono-hydroxyl and di-hydroxyl glucuronides, which were excreted in bile, not urine, with no remaining presence in plasma or tissues. Contrary to common assumptions, the majority of metabolites were excreted in bile. The novel integration method developed in this study utilizes the ratio of UV to mass response for metabolites within the same category, enabling the quantification of overlapping metabolite peaks. This novel approach proved to be very useful in the early preclinical stage of drug development without the use of ^14^C-labeled test compounds.

## 5. Future Research Directions

Lu AE90015 is a high-affinity negative allosteric modulator (NAM) of the human metabotropic glutamate 5 (mGlu5) receptor. It demonstrates a strong selectivity for mGlu5, with an over 1000-fold preference compared to the 79 other targets tested. The mGlu5 NAM mechanism is well characterized in preclinical studies and has shown therapeutic promise in clinical proof-of-concept trials for conditions such as fragile X syndrome, anxiety disorders, Parkinson’s disease dyskinesias, neuropathic pain, migraine pain, and gastroesophageal reflux disease (GERD). Pharmaceutical companies, including Novartis (Basel, Switzerland), AstraZeneca (Gaithersburg, MD, USA), Addex Pharmaceuticals (Sunrise, FL, USA) report advancing mGlu5 NAMs through phase I or II clinical trials for treating fragile X syndrome, L-DOPA-induced dyskinesias, neuropathic pain, migraine pain, and GERD. Lu AE90015 and its improved derivatives have the potential to be first-in-class drugs for treating these diseases. However, Lu AE90015 itself exhibits an extensive metabolism and high clearance, requiring further optimization. This study identifies metabolic labile sites of AE90015 that are suitable for structural modification and introduces an efficient, robust method for conducting subsequent metabolite studies.

## Figures and Tables

**Table 1:** Normalized molar amounts of AE90015 and its metabolites in relative molar amount in rats (*n* = 2) at time points during 24 h following oral administration of 30 mg/kg.

Integration/Molar Amount	AE90015	Hydroxyl-90015	Hydroxyl- Glucuronide	Di-Hydroxyl-90015	Di-Hydroxyl-Glucuronide	Excreted or Remaining in Body
Remain in Plasma	1.47%	2.15%	0.00%	2.15%	0.00%	5.77%
Remain in Liver	0.98%	0.49%	0.00%	0.85%	0.00%	2.32%
Remain in Brain	0.66%	0.05%	0.00%	0.00%	0.00%	0.70%
Remain in Kidney	0.16%	0.12%	0.00%	0.14%	0.00%	0.42%
Excrete from Urine	0.10%	1.86%	0.10%	23.65%	0.49%	26.19%
Excrete from Bile	0.39%	5.86%	5.57%	36.94%	15.83%	64.59%
Total Metabolites or Parent	3.26%	10.53%	5.67%	63.73%	16.32%	100.00%

## Data Availability

All data will be made available on reasonable request.
